# Energy-Level Interpretation of Carbazole Derivatives in Self-Assembling Monolayer

**DOI:** 10.3390/molecules29091910

**Published:** 2024-04-23

**Authors:** Raitis Grzibovskis, Arturs Aizstrauts, Anna Pidluzhna, Mantas Marcinskas, Artiom Magomedov, Smagul Karazhanov, Tadas Malinauskas, Vytautas Getautis, Aivars Vembris

**Affiliations:** 1 Institute of Solid State Physics, University of Latvia, LV-1063 Riga, Latvia; raitis.grzibovskis@cfi.lu.lv (R.G.); arturs.aizstrauts@cfi.lu.lv (A.A.); anna.pidluzhna@cfi.lu.lv (A.P.); 2 Department of Organic Chemistry, Kaunas University of Technology, 44249 Kaunas, Lithuania; mantas.marcinskas@ktu.lt (M.M.); artiom.magomedov@ktu.lt (A.M.); tadas.malinauskas@ktu.lt (T.M.); vytautas.getautis@ktu.lt (V.G.); 3 Department for Solar Energy, Institute for Energy Technology, 173 Kjeller, Norway; smagul.karazhanov@ife.no

**Keywords:** photoelectron emission spectroscopy, self-assembling monolayer, ionization energy, work function

## Abstract

Energy-level alignment is a crucial factor in the performance of thin-film devices, such as organic light-emitting diodes and photovoltaics. One way to adjust these energy levels is through chemical modification of the molecules involved. However, this approach may lead to unintended changes in the optical and/or electrical properties of the compound. An alternative method for energy-level adjustment at the interface is the use of self-assembling monolayers (SAMs). Initially, SAMs with passive spacers were employed, creating a surface dipole moment that altered the work function (WF) of the electrode. However, recent advancements have led to the synthesis of SAM molecules with active spacers. This development necessitates considering not only the modification of the electrode’s WF but also the ionization energy (IE) of the molecule itself. To measure both the IE of SAM molecules and their impact on the electrode’s WF, a relatively simple method is photo-electric emission spectroscopy. Solar cell performance parameters have a higher correlation coefficient with the ionization energy of SAM molecules with carbazole derivatives as spacers (up to 0.97) than the work function of the modified electrode (up to 0.88). Consequently, SAMs consisting of molecules with active spacers can be viewed as hole transport layers rather than interface layers.

## 1. Introduction

The utilization of thin-film devices, such as organic light-emitting diodes (OLEDs), photovoltaic (PV) cells, and field-effect transistors, has experienced significant growth in recent years, with some of these devices even reaching commercialization. These devices comprise multiple thin layers, either organic or inorganic, with each serving a specific purpose [1,2,3]. The selection of materials depends on the type of device and the desired function, whether it involves charge generation, light emission, or charge carrier transport. However, regardless of their specific functions, all these devices rely on the flow of electricity. Therefore, it is crucial to minimize resistivity in order to ensure optimal device performance.

The flow of electrons and holes is primarily influenced by two factors: the mobility of charge carriers in the material and the alignment of energy levels between the layers in the solar cell [4,5,6]. Both factors are dependent on the chosen compounds. The properties of the compounds can be changed by molecule structure modifications, which in turn change both the energy levels and the mobility of charge carriers [7,8]. However, it is challenging to modify each of these properties separately, and alterations in charge carrier mobility often lead to shifts in the energy levels of the molecule. This makes it difficult to create an efficient device with well-matched energy levels between the layers. Alternatively, the interface can be modified by using a self-assembling monolayer (SAM) [9,10,11]. SAMs are typically applied to the electrode, modifying its work function by increasing or decreasing it through the generated electric field. This reduces the energy barrier for charge carriers. To achieve this, the SAM molecules should possess a dipole moment to generate an internal electric field that facilitates the transfer of charge carriers through the interface [12,13]. Various types of molecules have been employed for such interface modification. SAM molecules usually consist of anchoring groups (-SH, -PO_3_H_2_, -COOH, -Si(OH)_3_, -COCl, and -PO_2_Cl_2_), which covalently bond to a modifiable layer; spacers (such as mercaptobi-phenyls, n-alkylene, p-phenylene, terpyrimidine, and phenylethynyl benzene); and terminal groups (H-, Cl-, CF_3_-, F-, Br-, and others) [14,15,16]. The spacer typically acts as a distance provider between the anchoring and terminal groups, thereby altering the dipole moment, and it does not actively participate in the transportation of charge carriers between the two layers. However, there exist SAM molecules with active groups like fullerene, tri-phenylamine, carbazole, and others, which function as spacers [17,18,19]. In this context, it is imperative to consider not only the impact of the dipole moment on the interface but also the ionization or electron affinity energy of the molecules. Consequently, an SAM can be perceived as a layer that serves as a conduit for either hole or electron transport, rather than merely a surface modifier.

Ultraviolet photoelectron spectroscopy (UPS) is the prevailing method employed to investigate the modification of the work function and ionization energy of SAMs. The cut-off binding energy is associated with the modification of the electrode’s work function, while the difference between the excitation photon energy and the width of the UPS spectrum provides the ionization energy of the SAM [20]. However, the UPS measurement system is relatively expensive and not very suitable for sample screening, as it requires high-vacuum conditions for measurement. In our previous work, we have demonstrated that photoelectric emission spectroscopy (PES) could serve as a viable alternative to UPS, as it has lower requirements for experimental systems [21]. UPS uses monochromatic UV radiation with a photon energy of 21.2 eV, and the kinetic energy distribution of the emitted electrons is measured. In the PES method, the photon energy is varied, and the number of emitted electrons depending on the photon energy is registered. As the photon energy is close to the ionization energy of the material, the kinetic energy of the emitted electrons is relatively low. This means that the scanning depth of PES can reach a couple of tens of nanometers, compared to 1–2 nm in the case of UPS [22].

This research paper delves into the investigation of six carbazole-based self-assembled monolayer (SAM) molecules. Our primary objective was to accurately measure the modified work function and ionization energy of these SAM molecules by using the photoelectric emission spectral dependence method. To the best of our knowledge, the utilization of the PES method for determining work function changes and ionization energy in a self-assembly monolayer has not been previously explored. Therefore, we conducted a comparative analysis of our results with the ionization energy of the bulk system formed by the SAM molecules, as well as scanning Kelvin probe (SKP) measurement of the electrode/SAM layer system. In addition to experimental measurements, we also performed quantum chemical calculations to determine the highest occupied molecular orbital (HOMO) and dipole moment of the molecules. These calculations were crucial to providing a comprehensive explanation for the results obtained from our experiments. Furthermore, we took our research a step further by establishing a correlation between the parameters obtained from the PES measurements and the performance of previously fabricated solar cells. This insightful analysis allowed us to gain a deeper understanding of the relationship between the measured parameters and the overall performance of the solar cells. By combining experimental measurements, quantum chemical calculations, and performance analysis, this research paper aims to contribute to the existing knowledge in the field of self-assembled monolayers and their potential applications in solar cell technology.

## 2. Discussion

**Figure 1:**
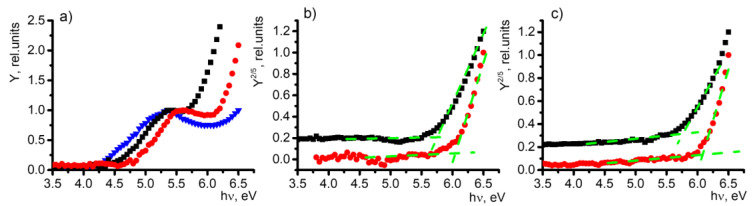
Photoelectron emission yield spectra of 2PACz (black squares), Cl-2PACz (red circles), and ITO (blue triangle) in (**a**) SAM films, (**b**) SAM films without ITO signal, and (**c**) bulky samples. Cross point of green lines represents the obtained energy.

An investigation of current dependence on irradiation photon energy reveals two distinct regions that exhibit a rapid increase in signal (see Figure 1a). The first surge is observed close to the energy corresponding to the work function of indium tin oxide (ITO), while the second surge at higher energy can be attributed to the photoelectron emission generated by the SAM compound. The advantage of the PES method is found in cases when the photoelectron emission signal originates from two separate sources, and the photoemission yield can be expressed as a combination of these distinct signals. In this particular scenario, the photoemission yield can be represented as follows:

(1) Y(hν)=x(hν−ΦITO)2H(hν−ΦITO)+y(hν−ISAM)52H(hν−ISAM),

where *x* and *y* are constants indicating the intensity of the signal originating from ITO and the SAM, respectively [23]; *hν* represents the photon energy; Φ*_ITO_* represents the work function of ITO; I*_SAM_* represents the ionization energy of the studied SAM; and *H*(E) is the Heaviside step function, with a value of *H*(E) = 0 for E < 0 and *H*(E) = 1 for E > 1 [23]. The aforementioned observation suggests the feasibility of isolating and examining the signals from each layer independently. Through the subtraction of the ITO-originating signal, we can extract the photoelectron emission spectrum exclusively generated by the SAM (see Figure 1b). Supplementary Information Figures S1 and S2 present the photoelectron emission yield spectra for all SAM compounds and bulk films, respectively.

The position of the initial signal increase is dependent on the self-assembled monolayer (SAM) that covers the electrode (see Figure 1a). Consequently, this change can be attributed to the alteration in the work function of the indium tin oxide (ITO) electrode. This demonstrates the practical application of a straightforward technique, namely, PES, in investigating self-assembled monolayers. It enables the identification of modifications of the electrode and provides valuable insights into the SAM energy levels. The modified work function for each SAM compound can be found in Table 1, along with the results of SKP measurements for the corresponding samples.

Observing the redistribution of charge carriers at the ITO/SAM interface, caused by the dipole moment of organic molecules, is possible through the measurement of the surface potential of the sample by using SKP. This measurement was conducted for both uncovered and SAM-covered ITO. In most cases, the results were found to be nearly identical, within the margin of error, to the PES results, providing evidence that the initial signal observed in photoelectron spectroscopy (PES) is indeed linked to changes in the work function of the ITO electrode. However, slight discrepancies (such as in the case of Cl-2PACz) can be explained by variations in experimental conditions. PES measurements were carried out in a vacuum while scanning Kelvin probe measurements were performed in an air environment.

In all cases, there is an increase in the work function, resulting in easier extraction of holes from the electrode. The molecules without halogen atoms as the terminal group, such as 2PACz and MeO-2PACz, show the smallest changes.

On the other hand, the molecules with halogen atoms, where the original 2PACz molecule was modified with electronegative atoms like F, Cl, Br, and I in the terminal group, have a significant impact on the shift in the work function. As the number of atoms in the terminal group increases, the work function also increases (see Table 1). However, the molecule with iodine is an exception, as it shows lower values.

The dipole moment directly influences the changes in the work function of the electrode. A higher dipole moment leads to larger changes. Interestingly, the molecule with iodine exhibits a similar dipole moment to F-2PACz, which explains why the work function changes are smaller for I-2PACz compared with the molecules with attached halogen atoms with a lower atom number.

In summary, the presence of halogen atoms in the terminal group and the dipole moment of the molecule play crucial roles in the shift in the work function of the electrode.

The ionization energy values of the materials examined in thin films were determined by analyzing the spectra of photoelectron emission. The threshold energy, which marks a significant increase in the yield of photoelectron emission, was utilized to calculate the ionization energy. As depicted in Figure 1c, the 2PACz and Cl-2PACz thin-film samples serve as an example.

**Figure 2:**
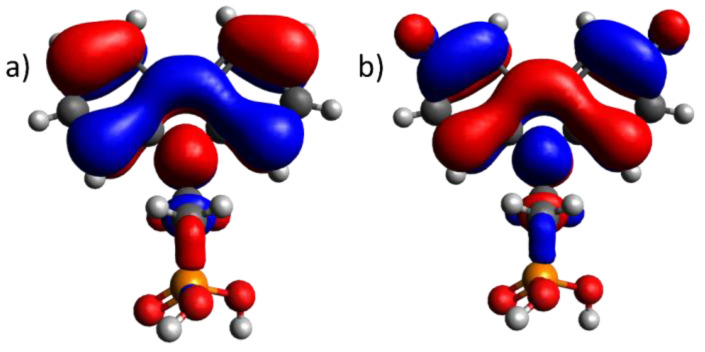
Highest occupied molecular orbital of (**a**) 2PACz and (**b**) F-2PACz. Ball colors represent atoms: white—hydrogen; gray—carbon; orange—phosphorus; red—oxygen. The colors of orbitals show the phase of the function. Blue stands for negative, and red stands for positive.

Upon comparing the ionization energy values obtained with the PES method with the highest occupied molecular orbital level of the molecules, a similar trend (see Table 1) that proves the electron origin from the highest occupied molecular orbital (ionization energy) was observed. The slight discrepancy between the ionization energy of the SAM and thin film can be attributed to measurement errors and molecule interactions in the case of thin films. However, no evidence of Fermi level alignment between the semiconductor and metal interface was detected, which would typically result in a shift in the ionization energy of the organic semiconductor [21,24]. This suggests that during the formation of a monolayer, there is no transfer of charge carriers, and the monolayer remains isolated from the electrode. Quantum chemical calculations further support this observation, as the HOMO is not present on the phosphonic acid group, which would typically form a covalent bond with ITO (refer to Figure 2 for 2PACz and F-2PACz). The highest occupied molecular orbitals for all compounds can be seen in Supplementary Information Figure S3.

The ionization energy level of 2PACz is initially measured at 5.75 eV. However, when the molecule is modified with halogen atoms, the ionization energy decreases to a range of 6–6.1 eV, as shown in Table 1. Generally, molecules with higher-electronegativity atoms tend to have higher ionization energy, with one exception being the fluorine atom, which has the highest electronegativity but the lowest ionization energy. The presence of oxygen at the same positions as the halogen atoms results in a decrease of approximately 0.6 eV in ionization energy.

**Figure 3:**
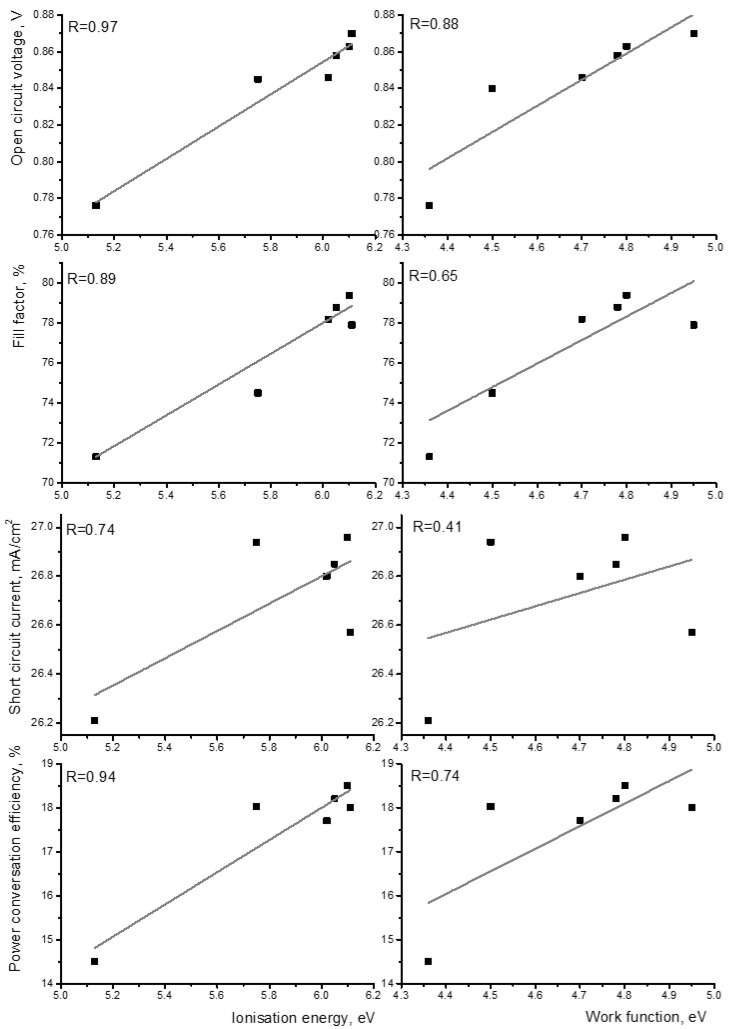
Correlation between energy levels and performance of solar cells. Right side—ionization energy of molecules; left side—work function of ITO. The correlation coefficient is represented in each graph.

The correlation (utilizing the correlation coefficient described in Supplementary Information) between the performance of solar cells and both the modified work function and ionization energy of self-assembled monolayer (SAM) molecules was calculated. The four performance parameters (open-circuit voltage, short-circuit current, fill factor, and power conversion efficiency) were utilized as performance indicators of the solar cells. The performance values of the solar cells were obtained from the literature, utilizing the same bulk heterojunction photoactive layer consisting of PM6 and BTP-eC9 [25,26,27]. The data from the solar cells are presented in Table S1. The ionization energy of the molecule exhibited strong correlation coefficients with the open-circuit voltage, power conversation efficiency, and fill factor of the solar cells of 0.97, 0.94, and 0.89, respectively (see Figure 3). All three parameters depend on the interface quality of the solar cells. Short-circuit current has the smallest correlation coefficient due to being possibly less impacted by the interface and more affected by the processes in the layer. In all cases, the correlation with the work function was found to be much weaker.

Based on these findings, it can be inferred that in the case of SAM molecules, where the active molecule derivative is located in the spacer, the ionization energy of this molecule plays a significantly more influential role in determining the device’s performance compared with the work function of the modified electrode. Consequently, such an SAM can be regarded as a hole transport layer.

## 3. Materials and Methods

### 3.1. Studied Materials

**Figure 4:**
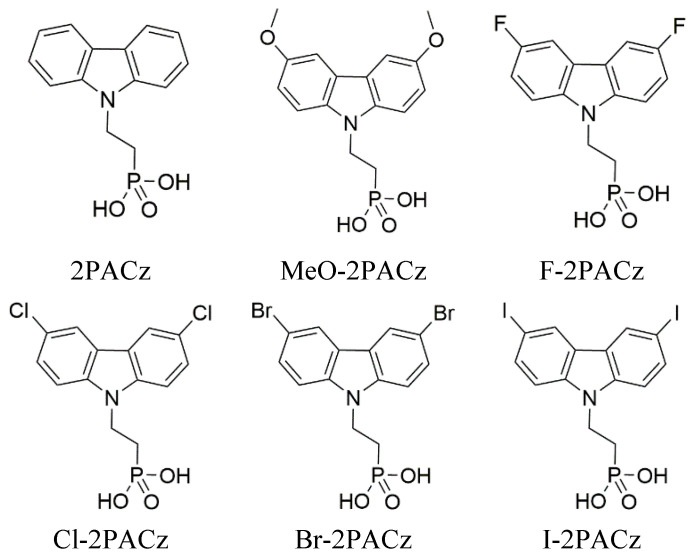
Studied materials and their chemical structures.

Six self-assembling monolayer materials were studied. The chosen materials are based on a carbazole core with an attached phosphonic acid anchoring group (see Figure 4) that binds to indium tin oxide (ITO) and fluoride, chloride, bromide, iodide, carbon, and methoxy as terminal groups. The synthesis procedure of these SAMs has been published previously [24,25,28].

### 3.2. Sample Preparation

Two sets of samples were prepared: monolayers and thin films. All of the films were deposited on ITO-covered glass substrates (20 Ω/sq; Präzisions Glas & Optik GmbH, Iserlohn, Germany) by using the spin-coating method.

To create the monolayers, the studied material was dissolved in tetrahydrofuran (THF) (Sigma Aldrich, St. Louis, MI, USA; 99.9%, inhibitor-free) at a concentration of 0.5 mg/mL. The spin-coating parameters were set as follows: rotation speed of 3000 rpm, acceleration of 3000 rpm/s, and rotation duration of 30 s. Subsequently, the samples were annealed on a hotplate at 100 °C for 10 min, and any excess material was washed off with THF. The thickness of the self-assembled monolayers (SAMs) was approximately 1 nm [29].

For the thin films, a solution was created in THF with a concentration of 5 mg/mL. The spin-coating parameters were adjusted to a rotation speed of 600 rpm, acceleration of 600 rpm/s, and rotation duration of 30 s. The samples were then dried on a hotplate at 100 °C for 10 min. The resulting films had a thickness of approximately 85–90 nm. The thickness was obtained by profilometer Profiler Dektak 150.

### 3.3. Film Characterization

In order to determine the ionization energy levels, the PES method within a vacuum environment of approximately 1 × 10^−5^ mBar was employed. The experimental setup consisted of the ENERGETIQ Laser-Driven Light Source (LDLS EQ-99) white light source, the Spectral Products DK240 1/4 m monochromator, and the Keithley 617 electrometer. The UV radiation was focused on a 5 × 5 mm^2^ area on the sample surface. By maintaining a distance of around 2 cm between the sample and the electrode responsible for collecting electrons, we ensured accurate measurements. Additionally, we applied a voltage of 50 V between the sample and electrode to amplify the obtained signal amplitude. Our measurement range spanned from 3.5 to 6.5 eV, with a precise step size of 0.05 eV. The power of UV radiation was from 6 µW/cm^2^ at 3.5 eV photon energy (354 nm wavelength) to 0.1 µW/cm^2^ at 6.5 eV photon energy (191 nm wavelength). In PES measurements, the photoemission yield *Y(hν)* can be calculated as

(2) $$Y\left(h\nu \right)=\frac{J\left(h\nu \right)}{P\left(h\nu \right)}$$

where *J(hν)* is the measured current (the number of emitted electrons) and *P(hν)* is the number of incident photons with the energy of *hν* [30]. The relation between photoemission yield and ionization energy (E_ioniz_) or work function can be expressed as a power law:

(3) $$Y\left(h\nu \right)=a{\left(h\nu -E_{ioniz}\right)}^{n}$$

where *a* is a constant showing the amplitude of the signal and *n* = 1 … 3 depending on the studied materials [31]. In the case of metals, *n* = 2 [30], while *n* = 2.5 … 3 is used in the case of semiconductors [32,33]. In this work, we used *n* = 2.5, as it gave a better approximation than *n* = 3. To obtain the ionization energy level of the material, *Y^1/n^(hν)* is calculated and its dependence on photon energy is plotted. The linear part of the *Y^1/n^(hν)* curve is extrapolated till *Y^1/n^(hν)* = 0. The obtained value is considered the ionization energy of the material.

To assess the work function of both ITO and ITO/SAM samples, we utilized the Kelvin probe Technology SKP5050. The vibration frequency of the probe was kept constant at 79 Hz. Highly oriented pyrolytic graphite (HOPG) was used as a reference material with the known work function of 4.93 ± 0.03 eV. To determine the absolute value of the ITO work function (WF), the difference between the measured surface potential of the HOPG sample (SP_HOPG_) and the surface potential of ITO (SP_ITO_) was subtracted from the known HOPG work function value:

(4) $$WF_{ITO}=4.93-\frac{SP_{HOPG}-SP_{ITO}}{1000}\;$$

As the system registers the surface potential in mV, the factor of 1000 in Equation (4) allows for the conversion of the data from mV to V.

The surface potential measurements with SKP were performed in air at room temperature. The data were obtained at ten different spots for each sample.

### 3.4. Quantum Chemical Calculations

Density Functional Theory (DFT) calculations were carried out by using the B3LYP functional together with a 6–31G(d,p) basis set. For molecules with iodine, C was used. The highest occupied molecular orbital energy and dipole moment of the molecules were calculated for optimized molecule geometry of the ground state. All calculations were carried out with the Gaussian 09W program [34]. Molecules and their respective HOMOs were drawn with the Avogadro program.

### 3.5. Solar Cell Performance Parameters

Solar cell performance can be characterized by four parameters: short-circuit current (J_sc_), open-circuit voltage (V_OC_), fill factor (FF), and power conversion efficiency (PCE). Short-circuit current is the current passing through the solar cell when the voltage across the solar cell is zero. Open-circuit voltage is the maximum voltage a solar cell can provide to an external circuit. Fill factor is the ratio of maximum obtainable power to the product of open-circuit voltage and short-circuit current. Power conversion efficiency is the percentage of the solar energy shining on a PV device that is converted into usable electricity.

## 4. Conclusions

In this study, we present the PES method as a highly effective tool for investigating both organic materials and the ITO/SAM interface simultaneously. Our findings reveal that the SAMs’ highest energy level closely aligns with the thin-film ionization energy, indicating a lack of Fermi level alignment at the ITO/SAM interface. Moreover, we observed that the presence of halogen atoms at the third and sixth positions in the carbazole moiety leads to an increase in the ionization energy of the molecule, while the presence of oxygen at the same positions results in a decrease in energy.

Furthermore, the low energy values obtained from PES measurements closely correspond to the values obtained through surface potential measurements by using SKP. These values are associated with the molecules’ dipole moment at the interface. It is worth noting that all SAMs were found to increase the work function of ITO, with the increase being directly proportional to the dipole moment of the molecules.

Importantly, our study highlights that the impact of SAM molecules’ ionization energy on device performance outweighs the modification of the electrodes’ work function by the SAMs. Therefore, SAM molecules that possess an active moiety in the spacer can be better characterized as hole transport materials.

## Figures and Tables

**Table 1:** Measured energy-level values of the studied materials on ITO, calculated energy of highest occupied molecular orbital, and dipole moment.

		Ionization Energy, eV			ITO Work Function, eV		
	Method	PES (Thin Film)	PES (SAM)	HOMO (eV)	PES	SKP	Dipole Moment (D)
Material							
ITO					4.30	4.30	
2PACz		5.75	5.74	5.42	4.50	4.41	1.89
MeO-2PACz		5.14	5.13	4.92	4.36	4.33	1.54
F-2PACz		6.04	6.02	5.52	4.70	4.66	3.78
Cl-2PACz		6.12	6.10	5.73	4.80	4.64	4.55
Br-2PACz		6.08	6.11	5.70	4.95	4.91	4.70
I-2PACz		6.06	6.05	5.95	4.78	4.75	3.86
		±0.03	±0.03		±0.03	±0.02	

## Data Availability

The data presented in this study are available upon request from the corresponding author.

## Supplementary Materials

The following supporting information can be downloaded at: https://www.mdpi.com/article/10.3390/molecules29091910/s1, Figure S1: Photoelectron emission yield spectra of SAM films, Figure S2: Photoelectron emission yield spectra of bulky samples, Figure S3: Highest occupied molecular orbital of SAMs, Table S1: Photovoltaic parameters of bulk heterojunction solar cells from references [25,26,27]. Voc—open-circuit voltage; FF—fill factor; Jsc—short-circuit current; PCE—power conversation efficiency.
