# Supplementary material for: Energy-Level Interpretation of Carbazole Derivatives in Self-Assembling Monolayer
Source: Molecules. 2024 Apr 23;29(9):1910. doi: 10.3390/molecules29091910 (PMC11085244; doi:10.3390/molecules29091910)
Supplement: Supplementary file 1 [file molecules-29-01910-s001.zip › molecules-2946273-supplementary.pdf]

Supplementary Information

Contents

Photoelectron emission yield spectra of SAM films……………………………………………1 pp

Photoelectron emission yield spectra of bulky samples……………………………………...4 pp

Highest occupied molecular orbital of SAM……………………………………………………7 pp

Photovoltaic parameters of bulk heterojunction solar cells…………………………………8 pp

Formula of correlation coefficient…………………………………………………………………...8 pp

References………………………………………………………………………………………………..8 pp

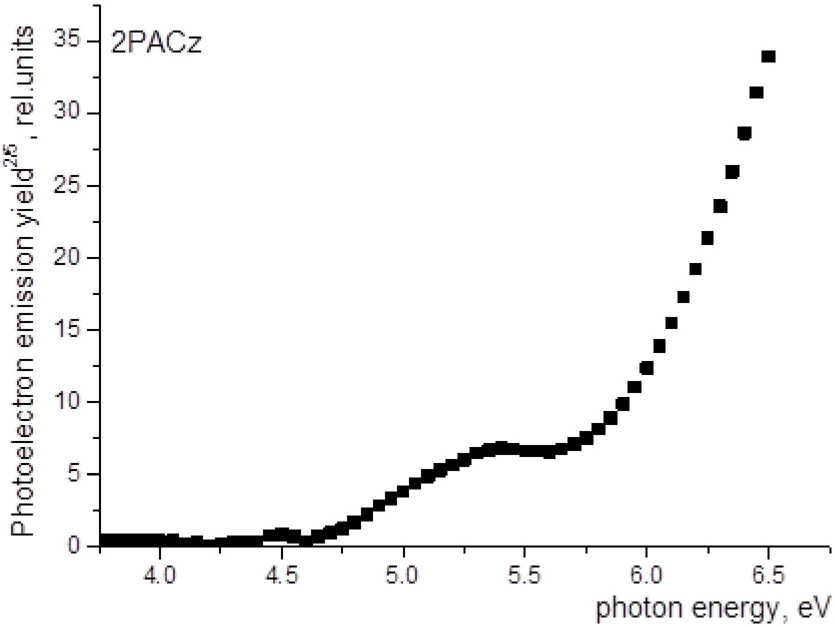

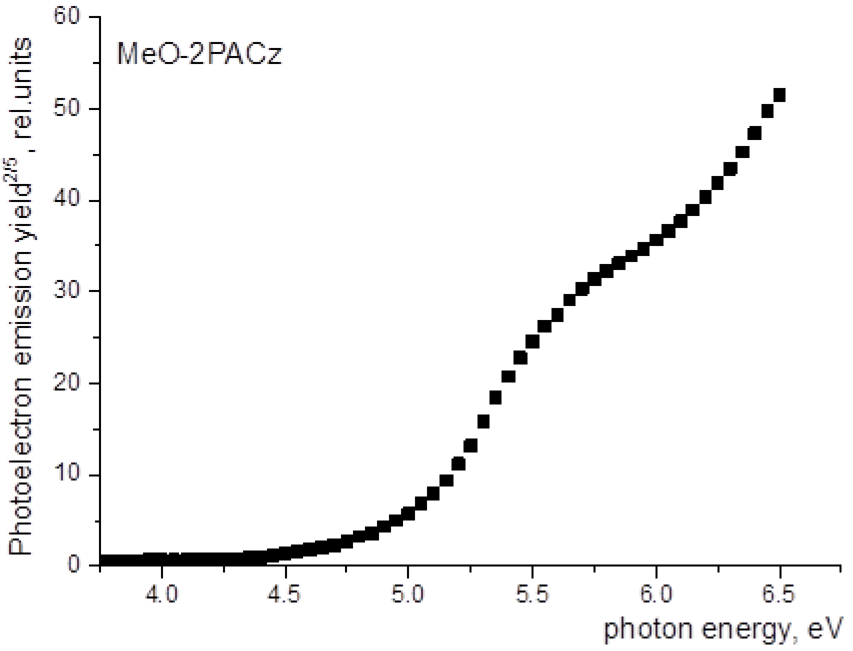
MeO-2PACz
Photoelectron emission yield$^{2/5}$, rel.units
photon energy, eV
0
10
20
30
40
50
60
4.0
4.5
5.0
5.5
6.0
6.5

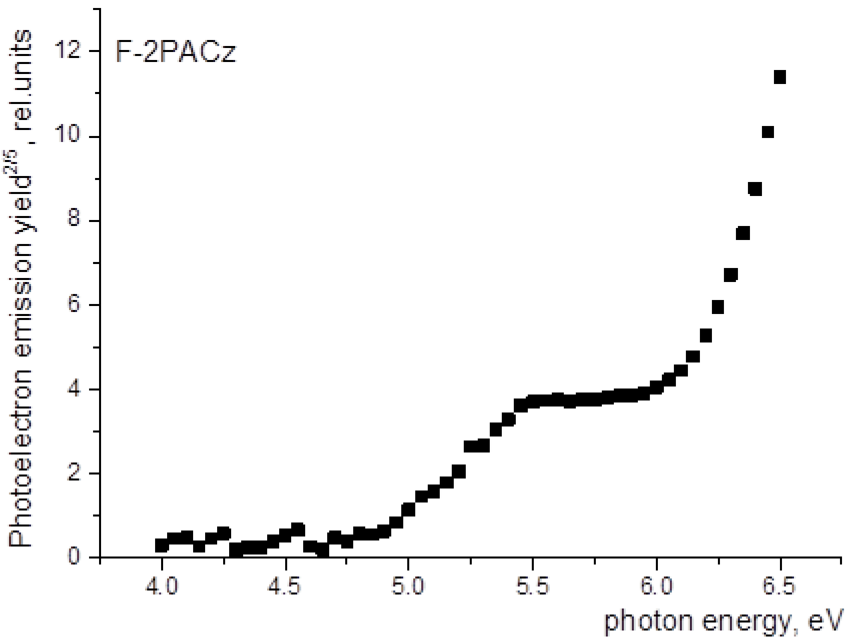
F-2PACz
Photoelectron emission yield$^{2/5}$, rel.units
photon energy, eV
0
2
4
6
8
10
12
4.0
4.5
5.0
5.5
6.0
6.5

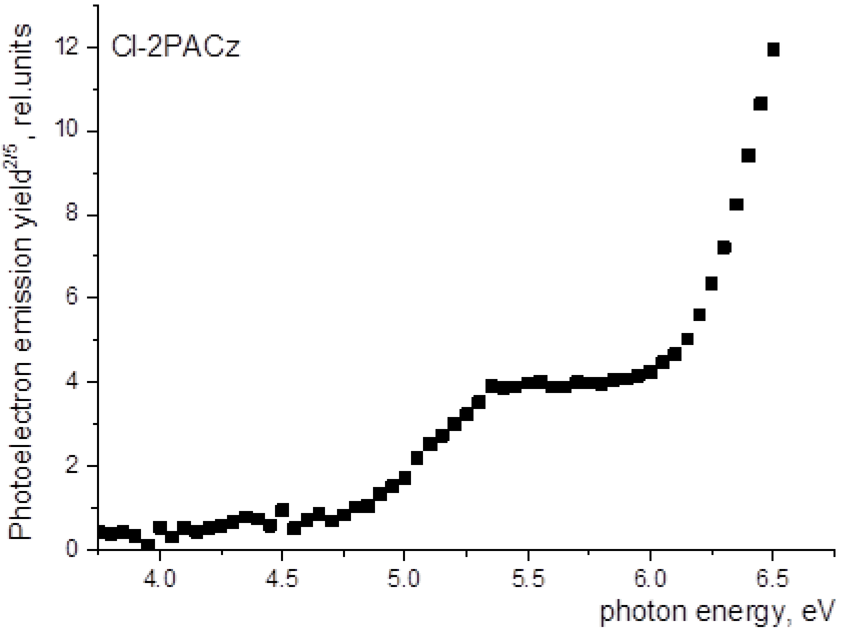
Cl-2PACz
Photoelectron emission yield$^{2/5}$, rel.units
photon energy, eV

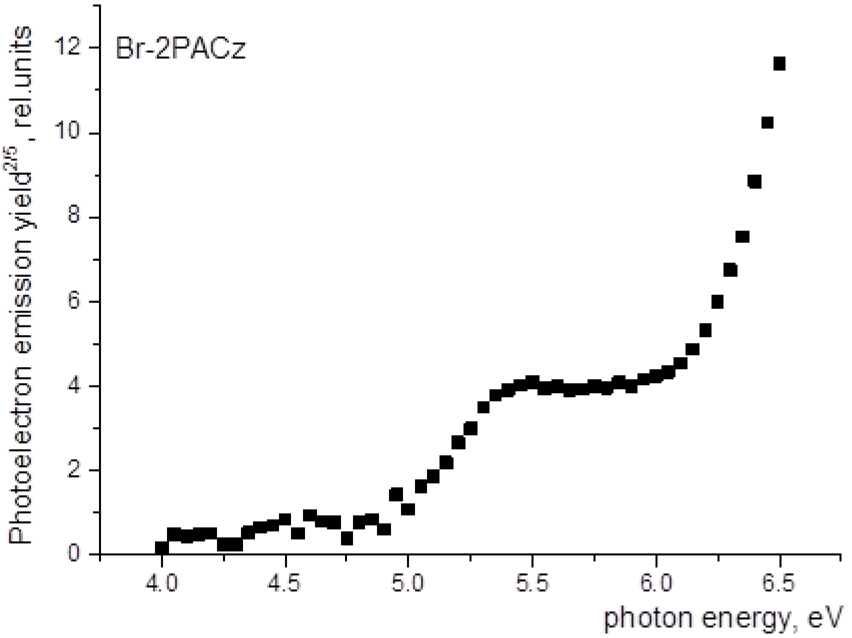
Br-2PACz
Photoelectron emission yield$^{2/5}$, rel.units
photon energy, eV

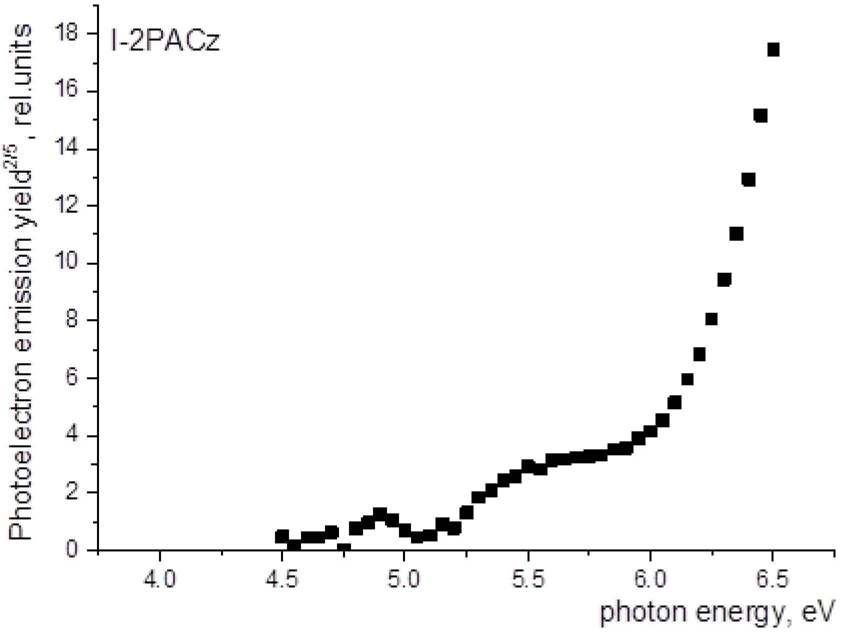

Figure S1. Photoelectron emission yield spectra of SAM films

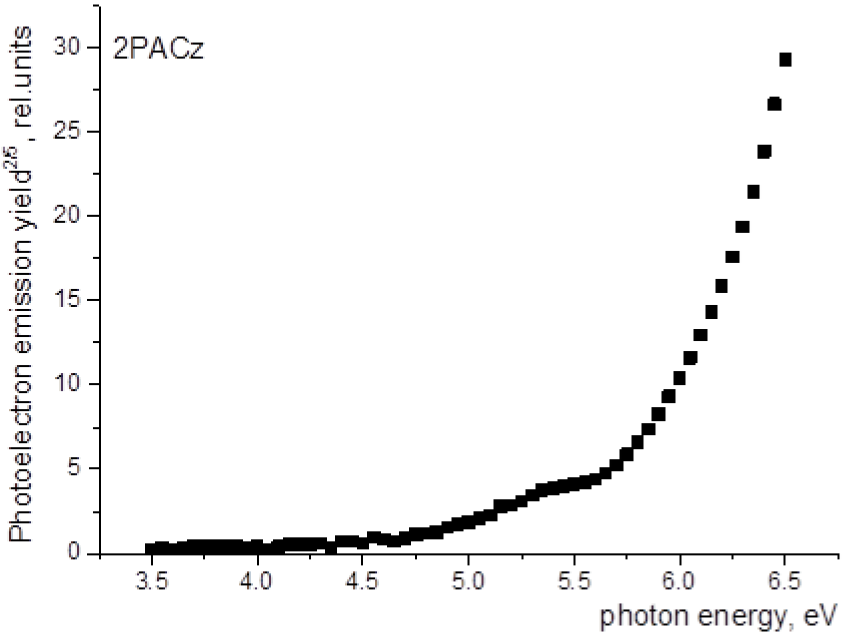

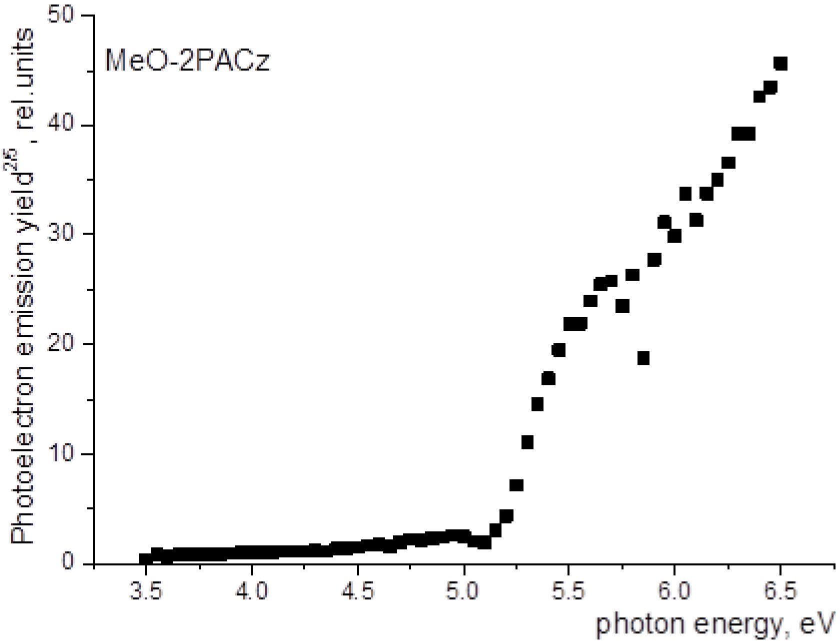
MeO-2PACz
Photoelectron emission yield^2/5, rel.units
photon energy, eV
0
10
20
30
40
50
3.5
4.0
4.5
5.0
5.5
6.0
6.5

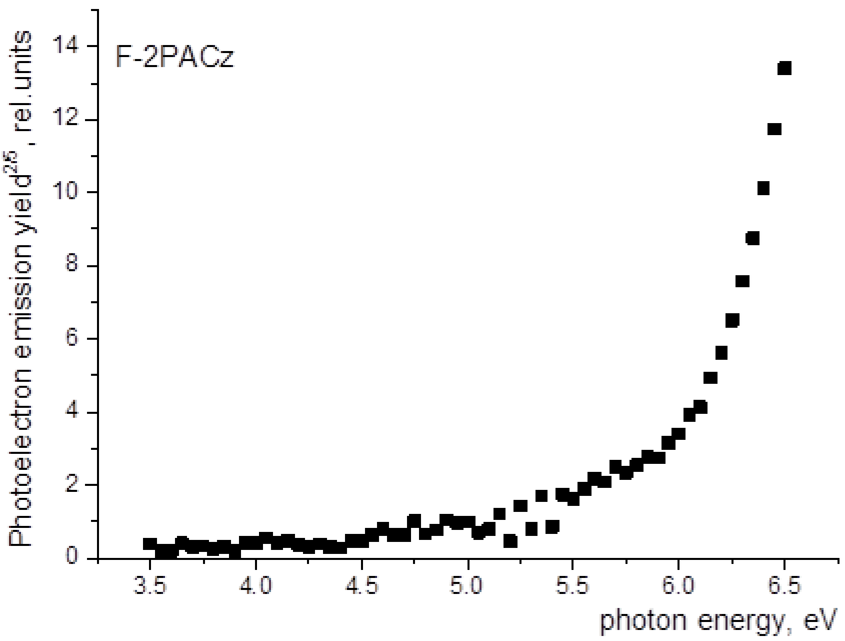
F-2PACz
Photoelectron emission yield^2/5, rel.units
photon energy, eV
0
2
4
6
8
10
12
14
3.5
4.0
4.5
5.0
5.5
6.0
6.5

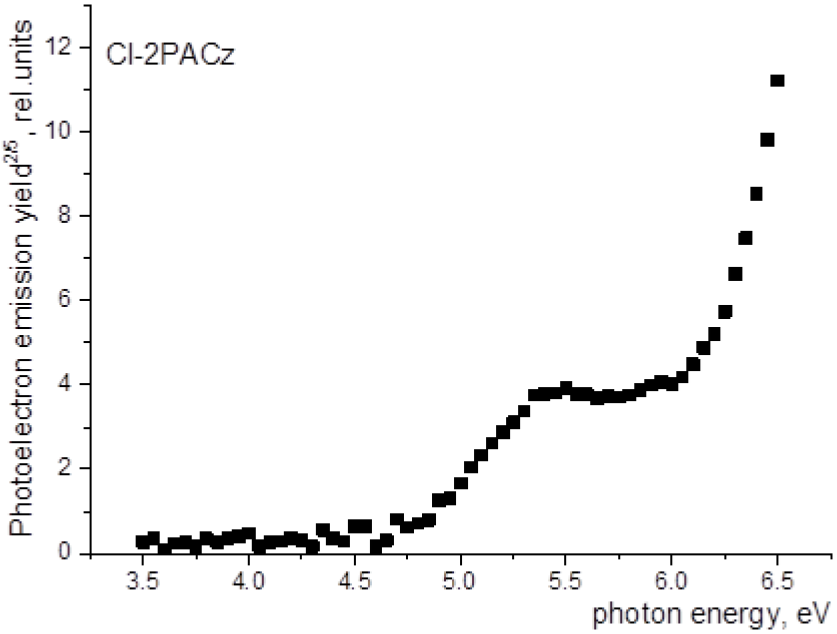

Cl-2PACz
Photoelectron emission yield$^{2/5}$, rel.units
photon energy, eV

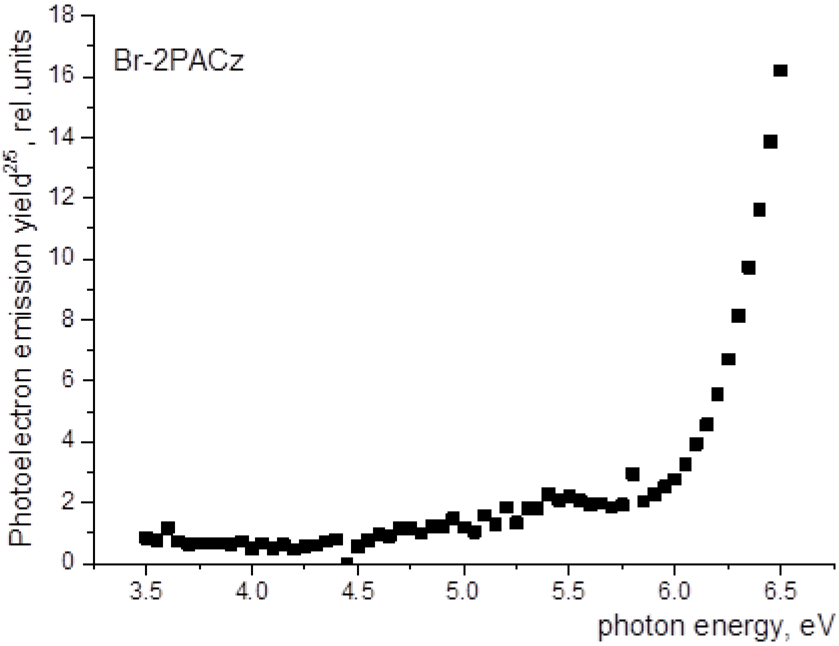

Br-2PACz
Photoelectron emission yield$^{2/5}$, rel.units
photon energy, eV

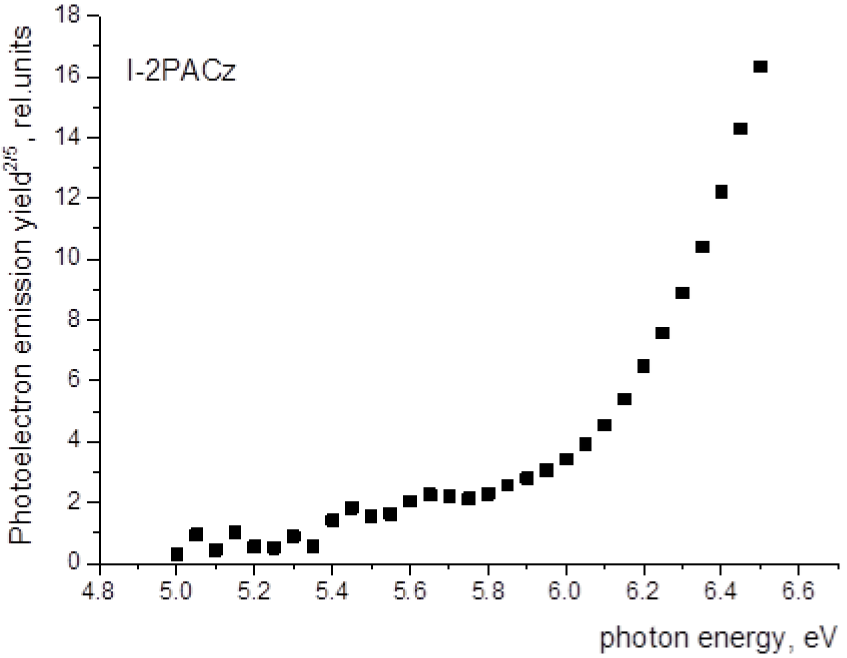

Figure S2. Photoelectron emission yield spectra of bulky samples

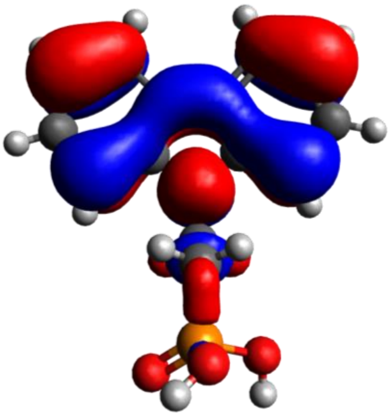

2PACz

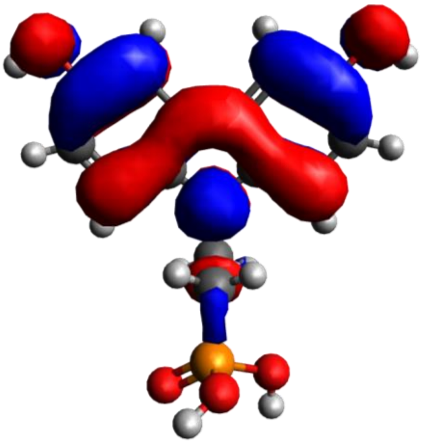

MeO-2PACz

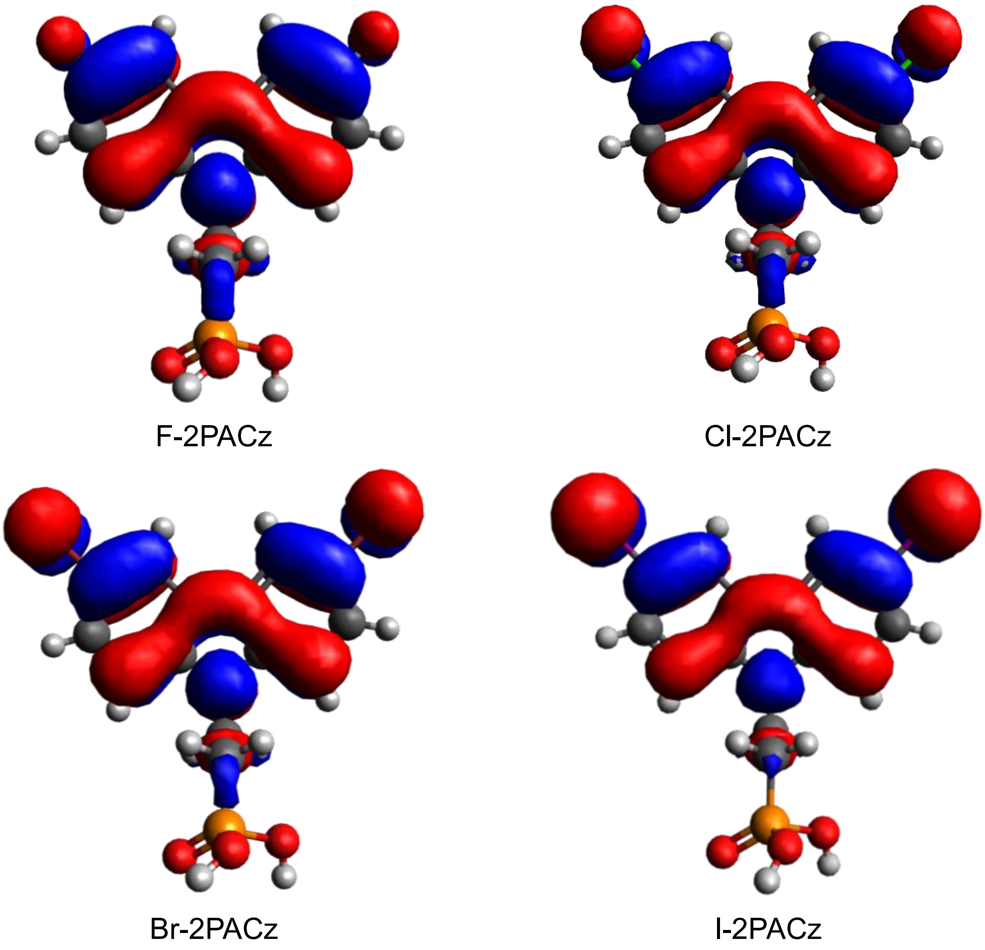

Figure S3. Highest occupied molecular orbital of SAM.

Table S1. Photovoltaic parameters of bulk heterojunction solar cells from references [25-27]. V_{oc} – open circuit voltage, FF – fill factor, J_{sc} – short circuit current, PCE – power conversation efficiency.

SAM	V_{oc}, V	FF, %	J_{sc}, mA/cm^2	PCE, %	References
2PACz	0.845	79.2	26.94	18.03	[1]
MeO-2PACz	0.776	71.3	26.21	14.5	[2]
F-2PACz	0.846	78.2	26.8	17.7	[3]
Cl-2PACz	0.863	79.4	26.96	18.5	[3]
Br-2PACz	0.870	77.9	26.57	18.0	[3]
I-2PACz	0.858	78.8	26.85	18.2	[3]

Correlation coefficient was calculated by the formula:

$$R = \frac{\sum_{i=1}^{n}(x_i - \bar{x})(y_i - \bar{y})}{\sqrt{\sum_{i=1}^{n}(x_i - \bar{x})^2}\sqrt{\sum_{i=1}^{n}(y_i - \bar{y})^2}},$$

where x – ionisation energy value or work function- value, y – solar cells parameter, $\bar{x}$ and $\bar{y}$ – mean values of the parameters.

References

[25] Lin, Y.; Zhang, Y.; Zhang, J.; Marcinskas, M.; Malinauskas, T.; Magomedov, A.; Nugraha, M.I.; Kaltsas, D.; Naphade, D.R.; Harrison, G.T.; et al. 18.9% Efficient Organic Solar Cells Based on n-Doped Bulk-Heterojunction and Halogen-Substituted Self-Assembled Monolayers as Hole Extracting Interlayers. Adv Energy Mater 2022, 12, doi:10.1002/aenm.202202503.

[26] Lin, Y.; Magomedov, A.; Firdaus Y.; Kaltsas, D.; El-Labban, A.; Faber, H.; Naphade, D.R.; Yengel, E.; Zheng, X.; Yarali, E.; et al. 18.4 % Organic Solar Cells Using a High Ionization Energy Self-Assembled Monolayer as Hole-Extraction Interlayer. ChemSusChem 2021, 14, 3569-3578, doi:10.1002/cssc.202100707

[27] Lin, Y; Firdaus, Y.; Isikgor, F.H.; Nugraha, M.I.; Yengel. E.; Harrison, G.T., Hallani, R.; El-Labban, A.; Faber, H.; Ma C.; et al. Self-Assembled Monolayer Enables Hole Transport Layer-Free Organic Solar Cells with 18% Efficiency and Improved Operational Stability. ACS Energy Lett. 2020, 5, 2935–2944, doi:10.1021/acsenergylett.0c01421
